# Evaluation of Mechanical Properties and Marginal Fit of Crowns Fabricated Using Commercially Pure Titanium and FUS-Invest

**DOI:** 10.1155/2017/5807304

**Published:** 2017-08-23

**Authors:** Jinshuang Wu, Xianli Wang, Helin Xing, Tianwen Guo, Chaofang Dong, Sefei Yang

**Affiliations:** ^1^Department of Stomatology, Chinese PLA General Hospital, Beijing 100853, China; ^2^Department of Stomatology, People's Hospital of Yuxi City, Yuxi 653100, China; ^3^Department of Prosthodontics, Anyang Sixth People's Hospital, Anyang 45500, China; ^4^State Key Laboratory of Military Stomatology, Department of Prosthodontics, School of Stomatology, The Fourth Military Medical University, Xi'an 710032, China; ^5^Institute of New Materials Technology, Beijing University of Science and Technology, Beijing 100853, China

## Abstract

This study investigated the mechanical properties and single crown accuracy of the tailor-made Fourth University Stomatology investment (FUS-invest) for casting titanium.* Background*. Current investment for casting titanium is not optimal for obtaining high-quality castings, and the commercially available titanium investment is costly.* Methods*. Titanium specimens were cast using the tailor-made FUS-invest. The mechanical properties were tested using a universal testing machine. Fractured castings were characterized by energy-dispersive spectroscopy. 19 titanium crowns were produced using FUS-invest and another 19 by Symbion. The accuracy of crowns was evaluated.* Results*. The mechanical properties of the titanium cast by FUS-invest were elastic modulus 125.6 ± 8.8 GPa, yield strength 567.5 ± 11.1 MPa, tensile strength 671.2 ± 15.6 MPa, and elongation 4.6 ± 0.2%. For marginal fit, no significant difference (*P* > 0.05) was found at four marker points of each group. For internal fit, no significant difference (*P* > 0.05) was found between two groups, whereas significant difference (*P* < 0.01) was found at different mark point of each group.* Conclusions*. The mechanical properties of titanium casted using FUS-invest fulfilled the ISO 9693 criteria. The marginal and internal fit of the titanium crowns using either the FUS-invest or Symbion were similar.

## 1. Introduction

Titanium and titanium alloys have unique biological and physical properties for dental application. These properties include excellent biocompatibility, high corrosion resistance, low thermal conductivity, little influence on magnetic resonance imaging, and mechanical properties comparable to type-IV dental gold alloys [1, 2]. Thus, titanium has been considered for dental applications such as removable partial dentures [3, 4] and fixed restorations [5, 6].

However, the conventional casting of titanium is difficult, due to its high melting point (1680°C) and highly reactive nature. At high temperatures, the titanium reacts to the mold components and oxygen and forms a reactive layer on the surface, termed the alpha-case layer [7, 8]. Components of the investment material that are in direct contact with the titanium may further contaminate the surface [9]. The oxide layer and mineral contamination change the surface properties and its bond strength to porcelain [10, 11]. Formation of the alpha-case layer changes the surface chemistry and microstructure of the castings, hence compromising mechanical properties, accuracy, durability, and fatigue resistance. In addition, the oxidized alpha-case layer is very hard and brittle and its removal is not straightforward; special techniques and equipment are required such as high pressure waterjets and laser ablation [12, 13]. Alternatively, formation of the alpha-case layer can be prevented using special machines for oxidation-resistant coatings and investments [14, 15].

Despite these issues related to high temperature, the traditional casting method is preferred over other fabricating techniques such as computer-aided design and computer-aided manufacture (CAD/CAM) and laser powder forming techniques such as selective laser sintering (SLS) and selective laser melting (SLM).

CAD/CAM technology is expensive compared with conventional casting and may not be suitable for mass production of crowns and bridges. Pure titanium has low machinability and shortens the tool life of machining burs [16, 17]. SLS/SLM is commonly used to fabricate titanium prostheses. The thermal heat transfer from the laser influences the molten pool size, thus affecting the geometrical tolerance of the built layers and accuracy [18], with titanium prostheses, oxidation, and powder adhesion to the titanium surface increasing roughness and compromising the quality of the marginal fit [19].

The conventional casting method and investments are commonly used in dental laboratories. During the casting, the dental investment materials physically contact the titanium at a high temperature that may affect the precision and mechanical properties of the casted titanium. This is particularly problematic for dental restorations where a high level of accuracy, internal fit (<73 *μ*m), and marginal fit (<100 *μ*m) are essential [20]. The alpha-case layer on the casted titanium surface increases not only the hardness and brittleness, but also the potential for fatigue, altered biocompatibility, segregation during solidification, and inhomogeneous microstructures [21, 22].

Casting shrinkage also directly influences the accuracy of the titanium castings. Although the setting and thermal expansion of the investment is likely to compensate for casting shrinkage, the high curing expansion is often uneven and may cause casting deformation. The thermal expansion of the material is mainly used to compensate for the shrinkage of titanium castings [22]. Therefore, the traditional investments generate a reactive layer on the surface of castings and are not optimal, considering the expansion of the casting mold. In addition, the commercially available titanium investments are costly.

Thus, it is important to develop new investment materials that will not interact with titanium at casting temperatures that are capable of fully compensating for shrinkage of the titanium casting and which are more economical. Our tailor-made dental investment material contained zirconium as a basic ingredient that hardly interacts with titanium. The present study investigated the viability of this tailor-made dental investment material for casting titanium, specifically the FUS-invest. This investment material using zirconium solution and organic binder can be completely decomposed following casting. There was little or no reaction between the base materials and the melt titanium. The following null hypotheses are tested in this study:

1. There is no difference between mechanical properties of titanium casting obtained from FUS-investment and the reference values of ISO standard.
2. There is no difference between castings made using the experimental and commercial investments.

## 2. Methods

**Figure 1:**
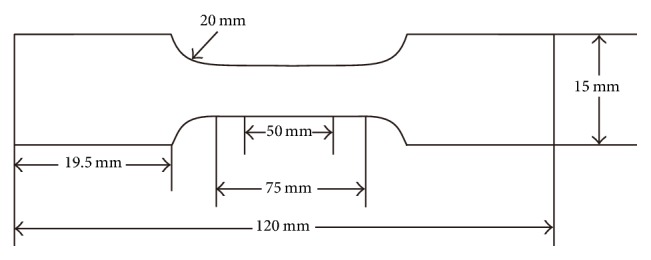
The dimensions of specimens for the tensile testing of titanium casts according to ISO 6892.

**Figure 2:**
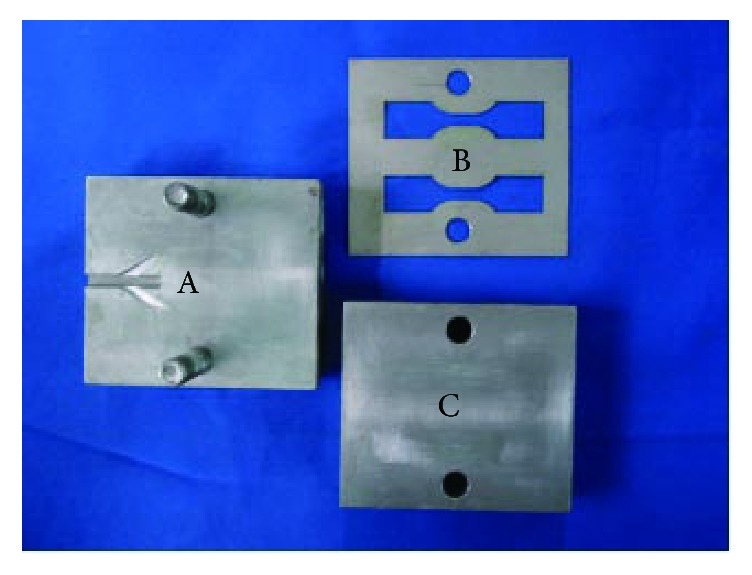
Molding die of specimen investment for strength. Wax perfusion-path; (B) specimen shape lining; (C) gland.

In accordance with the tensile testing standard (ISO6892), 7 dumbbell-shaped samples were prepared (Figure 1) of precise dimensions using a centrifugal titanium-vacuum pressure casting machine (LZ-2; Fourth Military Medical University of Oral Medicine and Luoyang Jian Xi General Machinery Factory, Xi'an, China): total length 120 mm; gauge length 50 mm; parallel length 75 mm; and thickness of specimen 0.8 mm. For each specimen, the wax patterns were prepared using casting wax (GEO Classic Transparent Blau; Renfert, Hilzingen, Germany) and stainless steel split die molds (Figure 2).

The prepared wax patterns were connected to sprues and cast using tailor-made FUS-invest zirconium-based investment, for dental crowns and bridges of cast titanium. This FUS-invest was prepared by mixing powder (ZrSiO_4_, 3Y-TZP, fused MgO, and titanium) and a zirconium solution (ZrOCl_2_·8H_2_O) in a ratio of 7.5 : 1 [23]. The casted commercially pure titanium (grade 2; Northwest Non-Ferrous Metal Processing Factory, Baoji, China) was abrasive-blasted (Jinan Baitong Sandblasting Machinery Factory, Jinan, China) with 50 *μ*m Al_2_O_3_ (East China Abrasive Apparatus, Taizhou, China), pressure 30 psi, at a distance 8 cm for 15 s. It was then cleaned in distilled water using an ultrasonic cleaner (Beijing Tai Tuo Precision Cleaning, Beijing, China) for 10 minutes and stored in a clean bag until tested further.

Prior to tensile testing, each specimen was inspected for visible cracks or defects and further checked for casting defects using an Industrial X-ray machine (MB320-13-5; SEFFER, Germany) set to 50 kV, 12 mA, and 4 s exposure time. Specimens with noticeably large pores over 0.7 mm or any defect within the range of the measuring length were excluded (one specimen) from further testing. The minimum dimension of the defect detected by this method was ~0.05 mm.

The resultant specimens (*n* = 6) with no defects such as cracks or inclusions and cavity within the range of the measuring length were used for tensile testing using a universal testing machine (Autograph AGS-10KNG; Shimadzu, Tokyo, Japan) at a crosshead speed of 0.5 mm/min until failure. The testing process was controlled by computer software (Trapezium 2, Version 2.1) and the elastic modulus *E*, yield strength *σ*0.2, tensile strength *σb*, and elongation *δ* were determined. Due to a measurement area of 10 mm^2^ to 100 mm^2^, values were rounded to the decimal.

The fractured sample was characterized using a scanning electron microscope (SEM; AMRAY-1000B; Amary, Delaware, USA). The fracture morphology was studied at ≥4 various points, including the edge of the fracture and the center of the sample. The elements of these 4 points were indexed by energy spectrum analysis using energy-dispersive X-ray (KYKY-Finder-1000; Noran, Massachusetts, USA).

**Figure 3:**
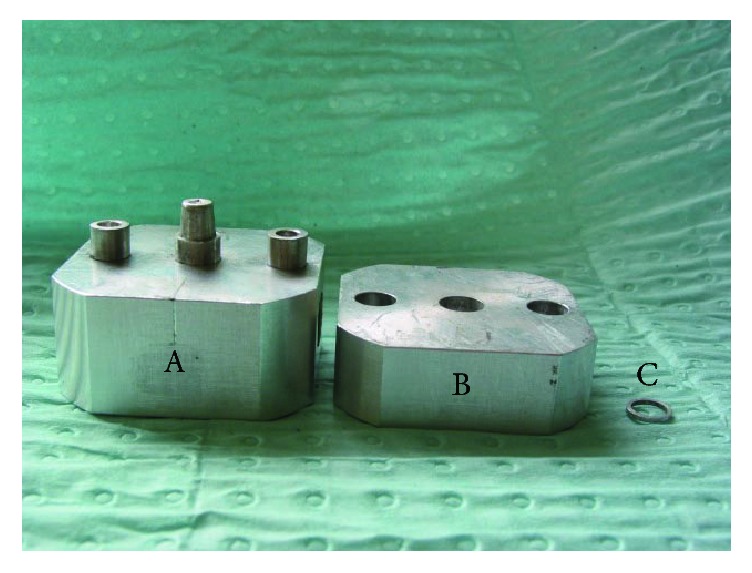
Molding die of single crown investment for accuracy test. (A) Molding die of standardized single crown; (B) gland; (C) stand-off.

**Figure 4:**
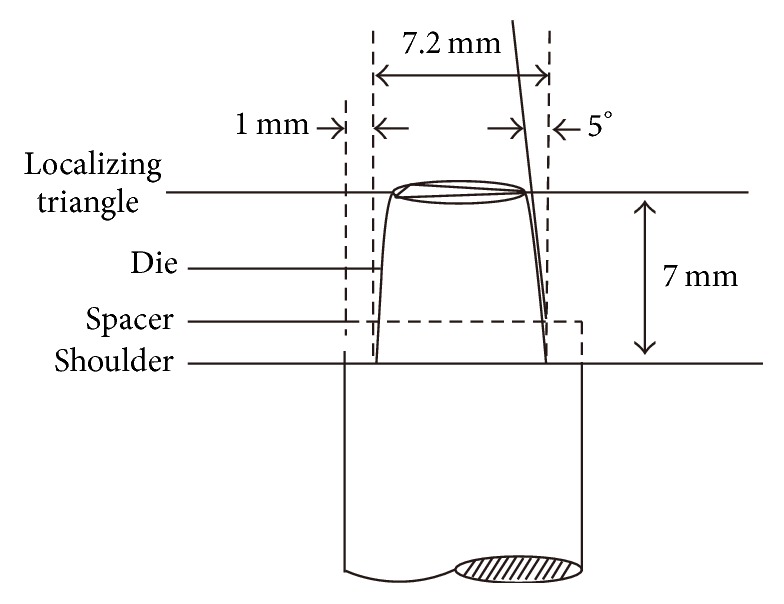
Molding die of standardized single crown.

**Figure 5:**
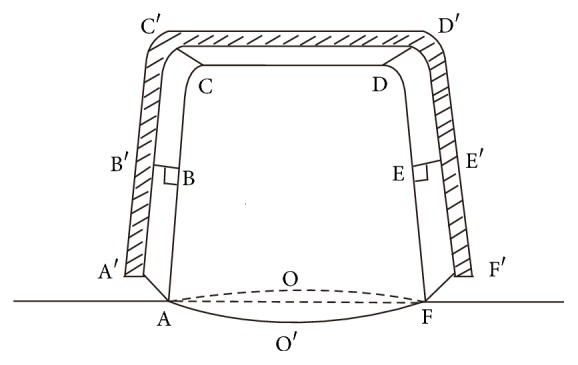
The marginal and internal mark points of the specimen (B, C, D, and E are the points of internal fit, and A, O, F, and O′ are the points of marginal adaptation).

For accuracy testing, an Aluminum alloy split molding die of single crown investment was used with an orientation triangle on top and a stand-off on shoulder (Figure 3). A stainless steel die with upper surface of 7.0 ± 0.1 mm, lower surface of 7.5 ± 0.2 mm, and a height of 6.5 ± 0.2 mm was used to obtain standardized wax patterns (*n* = 38) with a wall thickness of 0.5 mm using the drip wax method (Figure 4) [24]. The wax patterns were stored in plastic containers at room temperature for 48 hours to eliminate creeping. Four test points (A, O, F, and O′) were marked on the molding die (Figure 5). The wax patterns were positioned after removing the stand-off on the shoulder of the molding die about 1 mm. The distances from the wax patterns to the molding die at the 4 test points were each measured at 25x magnification using a measuring microscope (JLC 740033; Shanghai Optical Instrument Factory, Shanghai, China). Each point was measured 3x and averaged.

**Figure 6:**
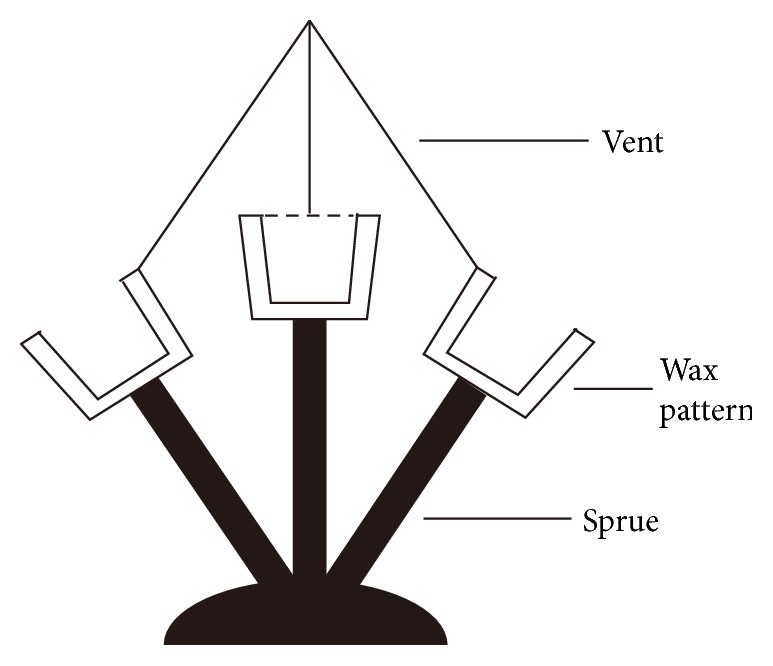
The erect method of perfusion-path of two groups.

To reduce the chances of deformation, the wax patterns were invested immediately after the measurement. The patterns were divided into two groups, the experimental group (*n* = 19), and the control group (*n* = 19). The experimental group comprised castings molds using FUS-invest zirconium-based investment and the control group consisted of commercially available titanium investments (Symbion TC; Nissin, Kyoto, Japan; Figure 6). Casting molds were made in accordance with the manufacturer's instructions.

The specimens were casted using the same centrifugal titanium-vacuum pressure casting machine and quickly cooled. After the investment was removed, the castings of both groups were polished using abrasive blasting (Al_2_O_3_ 50 *μ*m, pressure 30 psi, and distance 5 cm). The authors observed the inner face of the specimens using a magnifying glass (×3; Eschenbach, Xi'an Ogear Trade) to identify the quality of castings and presence of any artifacts. Any nodules that might prevent complete seating of the copings were removed with a round bur at low speed. Cleaning was performed with distilled water using an ultrasonic cleaner (Beijing Tai Tuo Precision Cleaning, Beijing, China) for 12 minutes.

According to the top localizing triangle, the test specimens were placed on the mold-die of a standardized single crown. A 15 N force was exerted on the occlusal surface by universal material-testing machine. The same handler was repeatedly measured at the reference points as before. The difference between the distance from the test specimens to the molding die and the distance from the wax patterns to the mold die was defined as the edge error *d*.

Each specimen of the two groups was equally cut into 2 parts along the axis with a linear cutting machine (DK 7725; Jiangsu Taizhou Changde Numerical Control Machine Factory, Jiangsu, China). One part was located on the molding die. A 15 N force was applied on the occlusal surface by the universal material-testing machine. On the molding die points B, C, D, and E were marked (Figure 5). The gaps between the inside of the specimen and the molding die at the marked points were measured by a measuring microscope (JLC740033, Shanghai). Each point was measured 3x and averaged. Each point was determined according to the influence of precision casting technology as shown in Figure 5.

### 2.1. Statistical Analysis

The data were analyzed by analysis of variance (ANOVA) and SNK for each group, and independent samples* t*-test was applied to compare the marginal and internal fits of the two groups at same mark point. All statistical analyses were performed using software (STATISTICA 17, Statsoft, USA; *α* = 0.05).

## 3. Results

### 3.1. Mechanical Properties of the Specimens

**Figure 7:**
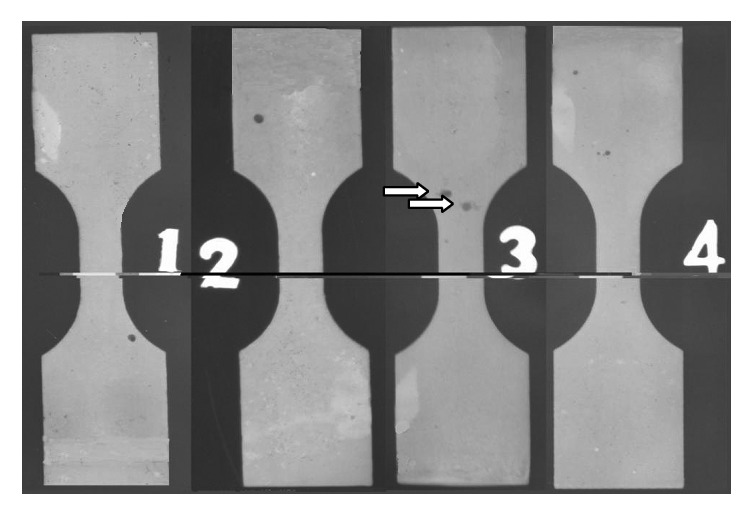
X-ray flaw detection figure of specimen for strength test (specimens with noticeably large pores (≥0.7 mm) or any defect within the range of the measuring length were excluded). The arrows refer to casting flaws.

The X-ray inspection of specimens did not show any bubble, tube hole, crack, or any other defect that could influence the tensile testing (Figure 7). Only one specimen (specimen #3) was excluded due to a visible defect (cloud-like patches; arrow).

The mechanical properties of the casting titanium using the FUS-invest zirconium-based investment (Table 1) showed elastic modulus (125.6 ± 8.8) GPa, yield strength (567.5 ± 11.1) MPa, tensile strength (671.2 ± 15.6) MPa, and elongation (4.6 ± 0.2)%. Both the marginal adaptation and internal fit of the two groups were similar (*P* > 0.05).

The mean thickness of the contamination layer was 74 *μ*m, which was measured from the surface of reaction to the titanium substrate.

**Figure 8:**
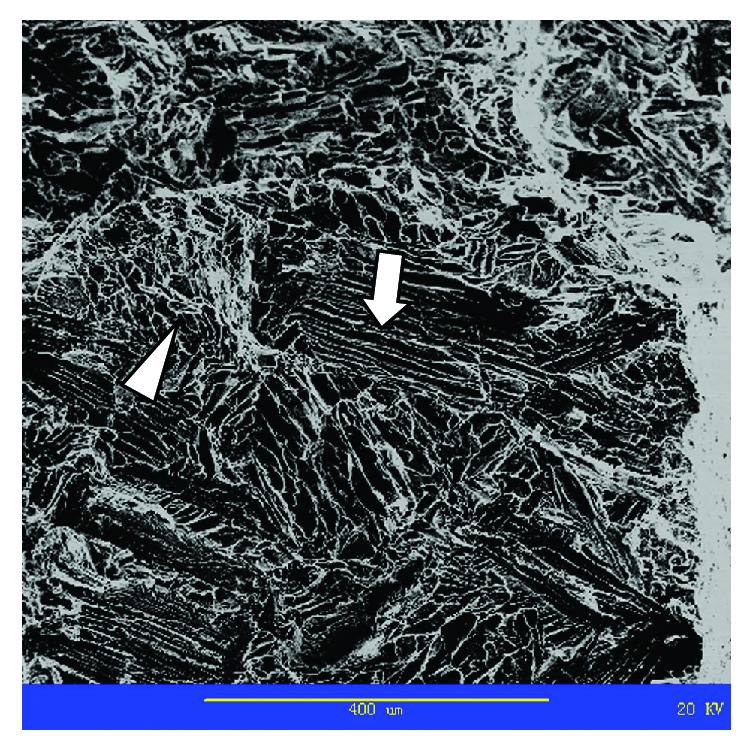
SEM Gray scale image of specimen fracture (arrow indicates location of striation, triangle indicates location of ductile dimple).

The SEM observation of the fracture surface (Figure 8) revealed mixed fractures, with typical dimples of ductile fractures and striation areas that are particular to brittle fractures. Typical ductile dimple morphology in the contamination layer was largely absent, but most of what we saw was a highlighted crystal boundary or phase boundary.

**Figure 9:**
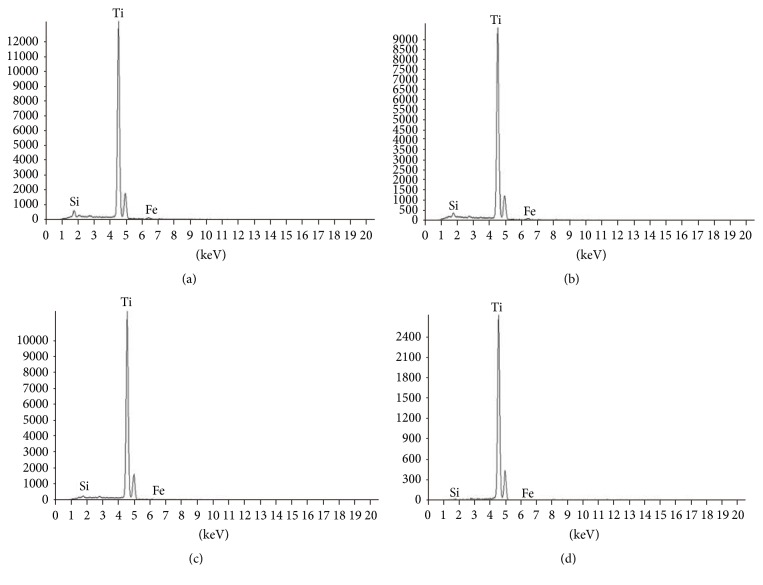
EDS analysis result of the fracture surface of specimen. (a) 13 *μ*m beneath. (b) 25 *μ*m beneath. (c) 50 *μ*m beneath. (d) 350 *μ*m beneath.

The X-ray energy-dispersive spectrometric (EDS) analysis of the fractured titanium (13 *μ*m from the surface) showed presence of extra elements, Si (5%) and Fe 1% (Figure 9). The Si and Fe were reduced to 3%, and 1%, respectively, at 25 *μ*m deeper from the surface. The Si was further reduced to 1% with zero Fe at 50 *μ*m deeper from the surface. The Si was only 0.6% and Fe was zero at a distance 350 *μ*m from the surface. The zirconium (Zr) was not found in the fractured surfaces.

### 3.2. Accuracy Comparison of Standard Single Crown

Examination of the specimens in each group showed a smooth surface free from cracks or metal nodules, with intact crown margins and no irregular burrs or pits.

**Figure 10:**
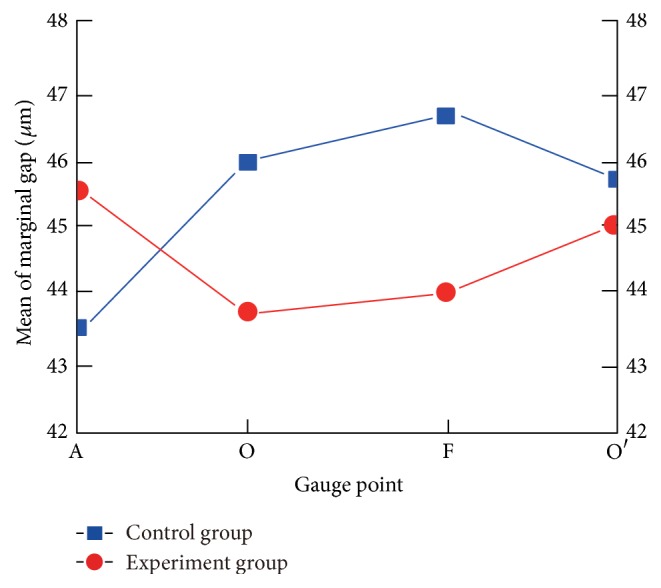
Mean of marginal adaptation in the experimental group and control group.

**Figure 11:**
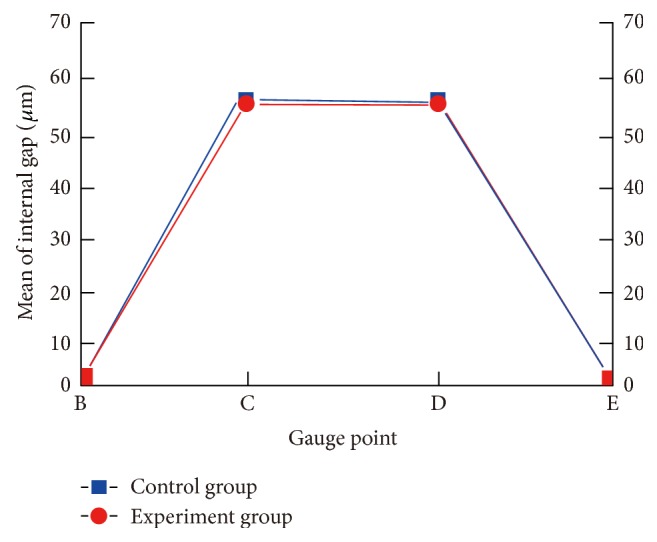
Mean of internal adaptation in the experimental group and control group.

The results of the marginal adaptation (Table 2, Figure 10) showed that the two groups were statistically comparable at each point (*P* > 0.05). There were also no significant differences at the 4 gauge points (A, O, F, and O′; *P* > 0.05, Table 3). The analysis of internal gaps at the different marks showed that there were no significant differences between the two groups at each point (*P* > 0.05; Table 4), but there was a significant difference in each group at different points (*P* < 0.01; Table 5, Figure 11). In each group, there was a significant difference at points B and E and at points C and D (*P* < 0.01; Table 6, Figure 11).

## 4. Discussion

The hypotheses that the mechanical properties of pure titanium casted using the FUS-invest can fulfil the demand of ISO without affecting the accuracy of crowns was validated.

The fabrication of titanium restorations by casting involves certain challenges and may affect the material properties (such as biocompatibility and strength) and marginal fit. The current study compared the effects of two investment materials on the accuracy of casted titanium crowns and investigated the mechanical properties of casted titanium using the FUS-invest. According to ISO standard 9693, the acceptable yield strength for alloys for fabricating metal-ceramic fixed partial denture frameworks is 250 MPa and the elongation threshold is 3%. ISO standard 6871 requires a higher yield strength threshold of 500 MPa for removable partial dentures [25]. Casted titanium has reportedly exhibited adequate mechanical properties, with tensile strength ~370 MPa–550 MPa, elastic modulus ~85 Pa–105 Pa, and elongation ~13%–20% [26–28]. Ibrahim et al. [26] reported the yield and tensile strength of commercially pure titanium casting as 535 MPa and 605 MPa, respectively. In the present study, the yield and tensile strengths of titanium specimens invested using FUS-invest were greater than those required by the ISO standard (Table 1), suggesting better resistance to deformation and longer clinical utilization. The elongation was lower than reported by Qiu et al. [27], which may be due to differences in thickness of the test specimens; the specimens used in the current study were thinner. Hence, with comparatively less elongation the reactive layer (or defects of casting) likely could promote greater brittleness and material failure.

The particular investment used for casting directly affects the thickness of the oxidation layer formed on the titanium surface. Guilin et al. [29] reported the thickness of the reaction layer for titanium castings associated with various investment materials: SiO_2_-based (~80 *μ*m), Al_2_O_3_-based (~50 *μ*m), and MgO-based (~14 *μ*m). In the current study, the thickness of the reaction layer in specimens cast using the FUS-invest was 74 *μ*m, which is thicker than that of the Al_2_O_3_- and MgO-based investments reported by Guilin et al. [29]. The specimens used by Guilin et al. [29] were ground to a smooth surface, polished, and acid-etched with a Keller solution, resulting in the partial removal of the reaction layers. However, for dental castings, the alpha-case layer cannot be removed, as it may alter the dimensions and precision required.

In the current study, the EDS analysis showed the presence of Si and Fe distributed in the fractured surface. The minim Si might be from zircon in investment, while the Fe might come from the abrasive blasting residue. The FUS-invest has two layers; the inner layer is surface material and there is no Si, and the outer layer is body material and there is some ZrSiO_4_ in order to decrease the cost without reducing the performance of the casting. The penetration of Si is particularly strong, so there is minim Si in the fracture surface. The FUS-invest material used a Zr solution and an organic binder that can be completely decomposed after casting. The base material hardly reacts to the molten titanium hence lacking Zr element in the reaction layer. Previous studies reported the incorporation of various elements in the casted titanium surface. For example, a SiO_2_-based investment may result in O and Si the casting; a Al_2_O_3_-based investment may result in O, Mg, and Al in the castings; and Mg-based investment materials may result in O, Mg, and Al in the castings [28, 30].

The marginal discrepancies and the internal gap of the crown may affect its success. A mean marginal gap of 100 *μ*m and internal gap of 73 *μ*m is considered clinically acceptable [31]. Our results indicated no significant differences in the marginal or internal gaps between the two investment groups. The marginal discrepancies of the experimental and control groups both ranged from 44 *μ*m to 46 *μ*m, which is well below the clinically acceptable limit.

These results show that the marginal discrepancies and the internal gaps of single crown casting using the FUS-invest can meet clinical demands and are comparable to the commercially available Symbion MgO-based titanium investment. While the internal gap between axial plane and axial angle was significantly different, the gap of axial plane was better than axial angle (Table 6, Figure 11). Perhaps the molten alloy setting leads to shrinkage of the castings along the axial wall, while the internal surface of the casting shows relative slip. In addition, the adaptation of the axial angle to the dye may be influenced by a number of factors, including the hardness, liquid metal flow, and the insufficient setting compensation. The precision of the single crown is also influenced by many factors, such as production mode, materials selection, and the design of the marginal configuration [32].

The quantity of industrial waste produced each year is increasing and has become a serious environmental and social issue. Current research on pure titanium investments concerns not only reducing the thickness of the reaction layer and increasing the precision of pure titanium castings, but also development of a reusable investment. The composition of the binder for these investments changes during the casting reaction or heat stress, which makes the cast difficult to reuse. Zhang et al. [33, 34] found that a binder-free dental investment, or investments with a binder containing soda-lime glass, could be reused. Concerning the FUS-invest material using zirconium solution and organic binder in the present study can be completely decomposed following casting. There was little or no reaction between the base material and the melt titanium, so the FUS-invest can be reused.

This study has a few limitations, as all results are based on the fabrication of single crown restorations. Results for long span bridges or cast dentures may differ, due to the greater quantity of titanium required. In addition, this study compared FUS-invest with only one commercially available investment for titanium castings. Comparisons with other investment materials for titanium casting may differ. Finally, the current study did not explore the bond strength for ceramic adhesion of titanium casted using FUS-invest, and this needs further research.

## 5. Conclusions

The mechanical properties of commercially pure titanium casted using FUS-invest materials fulfils the criteria described by the ISO 9693 and 6871 for the fabrication of fixed and removable partial dentures. The accuracy of the marginal and internal fit of the titanium crowns produced using the FUS-invest or Symbion MgO-based investment were not significantly different. The FUS-invest can meet the clinical demand and can be considered a cost-effective alternative to the Symbion MgO-based investment that is now commercially available.

## Figures and Tables

**Table 1:** Elasticity modulus, yield strength, tensile strength, and elongation of the specimens.

	Elasticity modulus *E*, GPa	Yield strength *σ*0.2, MPa	Tensile strength *σb*, MPa	Elongation *δ*, %
1	116.8	555.7	643.3	4.4
2	142.5	573.8	687.8	4.5
4	122.3	582.3	679.8	4.9

5	124.6	560.5	672.3	4.7
6	123.4	557.4	678.4	4.6
7	123.8	575.3	665.3	4.5

Mean ± SD	125.6 (8.8)	567.5 (11.1)	671.2 (15.6)	4.6 (0.2)

**Table 2:** Independent samples *t*-test of marginal adaptation on different marks.

	Levene's test for equality of variances		*t*-test for equality of means				
	*F*	Sig.	*t*	df	Sig. (2-tailed)	Mean difference	Std. error difference
The gap of point A	.018	.894	.119	36	.906	.78947	6.61718
The gap of point O	.017	.897	−.668	36	.508	−3.78947	5.67101
The gap of point F	.892	.351	−.481	36	.633	−3.00000	6.23259
The gap of point O′	.134	.717	−.281	36	.781	−1.52632	5.43670

**Table 3:** The analysis of marginal gap on different marks within group.

		Sum of squares	df	Mean square	*F*	Sig.
Control	Between different points	89.368	3	29.789	0.119	0.949
	Within same points	18074.316	72	251.032		
	Total	18163.684	75			

Experimental	Between different points	41.526	3	13.842	0.058	
	Within same points	17177.263	72	238.573		
	Total	17218.789	75			

One-way ANOVA was used for statistical analysis.

**Table 4:** Independent samples *t*-test of internal adaptation on different marks.

	Levene's test for equality of variances		*t*-test for equality of means				
	*F*	Sig.	*t*	df	Sig. (2-tailed)	Mean difference	Std. error difference
The gap of point B	.071	.791	−.385	36	.702	−.15789	.40994
The gap of point C	1.853	.182	−.294	36	.770	−.78947	2.68404
The gap of point D	.032	.858	−.094	36	.926	−.26316	2.80724
The gap of point E	1.413	.242	.372	36	.712	.10526	.28289

**Table 5:** The analysis of internal gap on different marks within groups.

		Sum of squares	df	Mean square	*F*	Sig.
Control	Between different points	58639.618	3	19546.539	813.943	0.000
	Within same points	1729.053	72	24.015		
	Total	60368.671	75			

Experimental	Between different points	56982.145	3	18994.048	665.536	0.000
	Within same points	2054.842	72	28.539		
	Total	59036.987	75			

One-way ANOVA was used for statistical analysis.

**Table 6:** SNK output of internal gap on different marks in experimental group.

	*n*	Subset for alpha = 0.05	
		1	2
Point E	19	0.5263	
Point B	19	0.7368	
Point D	19		55.2632
Point C	19		55.5263
Sig.		0.904	0.880
